# SVM-Fold: a tool for discriminative multi-class protein fold and superfamily recognition

**DOI:** 10.1186/1471-2105-8-S4-S2

**Published:** 2007-05-22

**Authors:** Iain Melvin, Eugene Ie, Rui Kuang, Jason Weston, William Noble Stafford, Christina Leslie

**Affiliations:** 1 NEC Laboratories of America, Princeton, NJ, USA; 2 Center for Computational Learning Systems, Columbia University, New York, NY, USA; 3 Department of Computer Science, UCSD, San Diego, CA, USA; 4 Department of Computer Science and Engineering, University of Minnesota, Minneapolis, MN, USA; 5 Department of Genome Sciences, University of Washington, Seattle, WA, USA; 6 Center for Computational Biology and Bioinformatics, Columbia University, New York, NY, USA

## Abstract

**Background:**

Predicting a protein's structural class from its amino acid sequence is a fundamental problem in computational biology. Much recent work has focused on developing new representations for protein sequences, called string kernels, for use with support vector machine (SVM) classifiers. However, while some of these approaches exhibit state-of-the-art performance at the binary protein classification problem, i.e. discriminating between a particular protein class and all other classes, few of these studies have addressed the real problem of multi-class superfamily or fold recognition. Moreover, there are only limited software tools and systems for SVM-based protein classification available to the bioinformatics community.

**Results:**

We present a new multi-class SVM-based protein fold and superfamily recognition system and web server called SVM-Fold, which can be found at . Our system uses an efficient implementation of a state-of-the-art string kernel for sequence profiles, called the profile kernel, where the underlying feature representation is a histogram of inexact matching *k*-mer frequencies. We also employ a novel machine learning approach to solve the difficult multi-class problem of classifying a sequence of amino acids into one of many known protein structural classes. Binary one-vs-the-rest SVM classifiers that are trained to recognize individual structural classes yield prediction scores that are not comparable, so that standard "one-vs-all" classification fails to perform well. Moreover, SVMs for classes at different levels of the protein structural hierarchy may make useful predictions, but one-vs-all does not try to combine these multiple predictions. To deal with these problems, our method learns relative weights between one-vs-the-rest classifiers and encodes information about the protein structural hierarchy for multi-class prediction. In large-scale benchmark results based on the SCOP database, our code weighting approach significantly improves on the standard one-vs-all method for both the superfamily and fold prediction in the remote homology setting and on the fold recognition problem. Moreover, our code weight learning algorithm strongly outperforms nearest-neighbor methods based on PSI-BLAST in terms of prediction accuracy on every structure classification problem we consider.

**Conclusion:**

By combining state-of-the-art SVM kernel methods with a novel multi-class algorithm, the SVM-Fold system delivers efficient and accurate protein fold and superfamily recognition.

## Background

Many statistical, homology-based methods have been developed for detecting protein structural classes from protein primary sequence information alone. These methods can be categorized into three major types: pairwise sequence comparison algorithms [1,2], generative models for protein families [3,4], and discriminative classifiers [5-9]. Many recent studies have shown that discriminative classifiers, such as support vector machines (SVMs), outperform the other two types of protein classification methods [10] in the context of binary remote homology detection – prediction of whether a sequence belongs to a single structural class or not – especially when incorporating unlabeled protein data [11,12]. However, it is uncertain how to combine these predictive binary classifiers properly in order to tackle the multi-class problem of classifying protein sequences into one of many structural classes.

In this work, we present the SVM-Fold protein classification web server and software package, which combines a state-of-the-art string kernel based on protein sequence profiles [13] with a novel multi-class classification algorithm designed for the protein structural hierarchy. We outline the main machine learning ideas behind our multi-class approach, which we call adaptive codes, and present large-scale benchmark experiments based on the SCOP hierarchy [14] for two difficult protein classification problems: remote homology detection and fold recognition. The SVM-Fold server is trained to perform multi-class SCOP fold and superfamily recognition and is available at [15].

In the machine learning literature, two main strategies have been devised to tackle multi-class prediction. The first strategy is to train a "single machine" to directly produce multi-class outputs. This approach includes multi-class formulations of the well-known binary support vector machine optimization problem [16,17]. However, complex multi-class optimization problems are often computationally expensive and impractical when the number of classes is large. A second and more tractable strategy is to solve a set of binary classification problems and process the binary predictions to obtain a multi-class prediction [18,19]. This approach assigns to each test example a vector of real-valued discriminant scores or binary prediction rule scores, which we call the *output vector *for the example. This second class of methods includes widely-used approaches such as one-vs-all, all-vs-all, and error-correcting output codes. In the standard one-vs-all approach, one trains *N *one-vs-the-rest classifiers to obtain a length-*N *output vector and then predicts the class with the largest margin. Standard all-vs-all is similar, except that one trains all pairwise binary classifiers to obtain a length *N*(*N *- 1)/2 output vector [18]. One can also represent different classes by binary vectors or *output codes *in the output vector space (We will use the terms "output space" and "code space" interchangeably for the output vector space.) and predict the class based on which output code is closest to the binary output vector for the example [19,20]. While this approach, called error-correcting output codes (ECOC), generalizes the other standard methods, a recent empirical study suggests that the one-vs-all approach performs as well or better in most cases [21]. One failing of one-vs-all is that it assumes that the margins of the component binary classifiers are comparable, so that the individual classifier with the largest prediction corresponds to the best class. This assumption is often invalid in practice and in particular for SVM classifiers, since SVM prediction scores are not probabilistic and cannot be readily interpreted aa class conditional probabilities or converted to *p*-values. One proposed method for computing probabilities from SVM outputs is to fit a sigmoid function to the predicted margins for each classifier [22]. After this procedure, the output probabilities rather than the margins are compared in one-vs-all. However, in many applications, the training data may be insufficient to fit the sigmoids accurately, or the sigmoids may be poor models for the margin distributions. Moreover, one-vs-all and the other standard output vector approaches do not take advantage of known relationships between classes, such as hierarchical relationships in the protein structural taxonomy. Here, we present a simple but effective multi-class method for protein fold recognition that combines the predictions of state-of-the-art one-vs-the-rest SVM protein classifiers by learning in the output space. In order to solve the problem that prediction scores from different classifiers are not on the same scale, we solve an optimization problem to learn a weighting of the real-valued binary classifiers that make up the components of the output code. Instead of using *ad hoc *or random output codes as in ECOC, we design codes that are directly related to the structural hierarchy of a known taxonomy, such as the manually curated Structural Classification of Proteins (SCOP) [14], with components that correspond to fold and superfamily detectors. In large-scale benchmark experiments based on SCOP, we find that our adaptive codes method significantly outperforms one-vs-all in both the remote homology detection and the fold recognition setting. Moreover, we find that our method successfully exploits hierarchical information in the remote homology setting, in that multi-class prediction performance continues to increase as we add more code elements corresponding to more levels of the SCOP hierarchy. We also dramatically outperform a nearest-neighbor approach based on PSI-BLAST in terms of multi-class prediction accuracy for every structural classification problem that we consider.

The current work is an expanded version of a conference proceedings paper [23], with larger scale experiments of the adaptive codes method. This work also describes our web server implementation, SVM-Fold, which is available for use at SVM-Fold [15]. A companion paper that focuses more on the algorithmic details of code learning will appear elsewhere.

## Results and discussion

### Remote homology detection and fold recognition in a multi-class setting

In this work, we focus on two protein classification problems that have long been studied but are still considered unsolved: remote homology detection and fold recognition. In remote homology detection, we wish to recognize when a new protein sequence has a distant evolutionary relationship to a protein sequence in a database (e.g., one whose structure is known). Due to a distant common ancestor, the protein sequences exhibit subtle sequence similarities (remote homology) that cannot generally be detected by statistical, alignment-based methods [1,24]. In fold recognition, we wish to recognize when a new protein sequence will exhibit the same fold as a protein from the structure database, even if is there is no evidence of any evolutionary relationship between the proteins.

**Figure 1:**
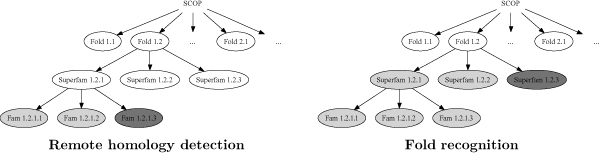
**Two protein classification problems**. (Left) In the SCOP database, we simulate the remote homology detection problem by holding out a test family (shown in dark gray) from a superfamily and using the other families as positive training data (shown in light gray). The task is to correctly predict the superfamily or fold membership of the held-out sequences. (Right) We simulate the fold recognition problem by holding out a test superfamily (dark gray) from a fold and using the other superfamilies as training data (light gray). The task is to correctly recognize the fold of the held-out sequences.

We base our experiments on SCOP, a manually curated hierarchical classification system for known protein structures. At the top level of the hierarchy are SCOP very broad structural classes. These are subdivided into folds, consisting of sequences that have the same general 3D structural architecture. SCOP folds are divided into superfamilies, containing sequences that are at least remotely homologous (evolutionarily related). Finally, each superfamily is further divided into families, consisting of homologous sequences with an easily detectable level of sequence similarity. We can design experiments based on the SCOP hierarchy to test performance on both the remote homology detection and the fold recognition problem, as depicted in Figure 1.

To test our final fielded system, we assembled benchmark data sets for the remote homology detection and fold recognition problems using sequences from the SCOP 1.65 protein database. We used ASTRAL [25] to filter these sequences so that no two sequences share greater than 95% identity. Note that the actual database used on the web server differs from these datasets, as there is no requirement for a test set.

### Benchmark data sets

For the fold recognition problem, we designed our benchmark experiments so that the test set consists of held-out superfamilies belonging to folds that are represented in the training data. We prepared a data set by first removing all superfamilies that have less than 5 sequence examples. We then removed all folds that have less than 3 superfamilies. We selected superfamilies for testing at random from the remaining superfamilies such that the test set for the superfamily contains no more than 40% of the remaining sequences for the fold. If at least one suitable superfamily could not be found, then the fold was removed from the experiment. The resulting fold detection data set contains of 26 folds, 303 superfamilies, and 652 families for training. We completely hold out 614 sequences from 46 superfamilies for testing.

For the remote homology detection, the test set should contain held-out families belonging to superfamilies that are represented in the training data. One can evaluate performance for multi-class prediction of fold or superfamily levels, and it is natural to try different codes for these two tasks; therefore, we prepared a separate data set for remote homology superfamily and fold detection. For the superfamily data set, we used the same selection scheme as for fold recognition, except the minimum number of sequences for the children of the superfamilies is relaxed to 3, and we selected random families for testing instead of superfamilies. The resulting superfamily detection data set contains of 74 superfamilies, and 544 families for training. We completely hold out 802 sequences from 110 families for testing.

For the remote homology fold detection data set, we first removed all superfamilies with less than 2 families. We then selected families from the remaining superfamilies for testing. We selected families at random from each superfamily such that we never selected more than 40% of the parent superfamily for testing. If no such families were found then the superfamily was removed from the data set. If a fold was then found to have no superfamilies with held out families for testing, it was removed from the data set. The resulting remote homology detection set contains 44 folds, 424 superfamilies, and 809 families for training. We completely hold out 381 sequences from 136 families for testing.

When training base classifiers, we only use negative data from outside of the target class of the experiment. For fold recognition, this means that when we train superfamily or family detectors, we exclude negative example sequences that come from the parent fold.

### Training workflow for benchmark experiments

**Figure 2:**
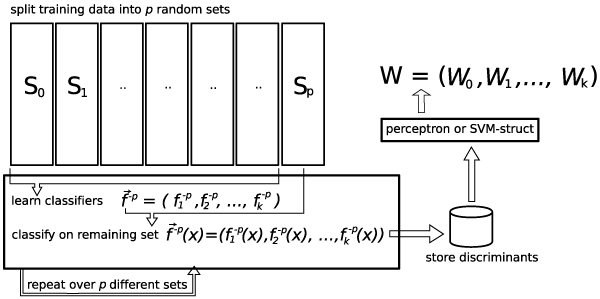
**Summary of the main stages of the adaptive code methodology for multi-class protein classification**. The figure gives an overview of the training workflow for the adaptive codes method. The box for learning weights is illustrated in detail in Figure 3.

**Figure 3:**
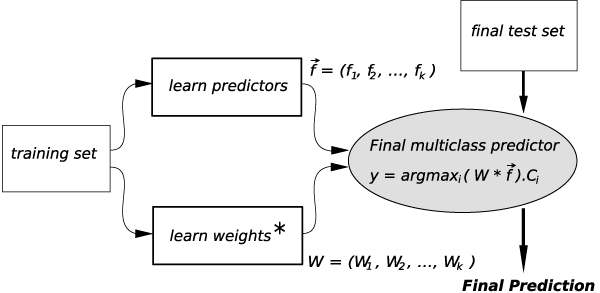
**The mechanism for learning code weights**. The figure shows the cross-validation set-up for supervised learning of the weight vector **W**.

To set up the adaptive codes optimization problem during training, we use a cross-validation scheme to embed protein sequences in an output space, representing each protein as a vector of SVM discriminant scores. Suppose the number of superfamilies and folds in a SCOP-based data set is *k *and *q *respectively, and suppose we are interested in codes that incorporate fold and superfamily levels of the hierarachy, for example. Then our approach for solving the multi-class protein classification problem involves producing a real-valued output vector f→
 MathType@MTEF@5@5@+=feaafiart1ev1aaatCvAUfKttLearuWrP9MDH5MBPbIqV92AaeXatLxBI9gBaebbnrfifHhDYfgasaacH8akY=wiFfYdH8Gipec8Eeeu0xXdbba9frFj0=OqFfea0dXdd9vqai=hGuQ8kuc9pgc9s8qqaq=dirpe0xb9q8qiLsFr0=vr0=vr0dc8meaabaqaciaacaGaaeqabaqabeGadaaakeaacuWGMbGzgaWcaaaa@2E13@(*x*) = (*f*_1_(*x*),..., *f*_*k*+*q*_(*x*)) for each test sequence *x*, where the *f*_*i *_are binary SVM superfamily or fold detectors trained using profile string kernels [11], and using (*k *+ *q*)-length code vectors **C**_*j *_that encode the superfamily and fold of a protein class as a bit vector. We use training data to learn a weight vector **W **= (*W*_1_,...,*W*_*k*+*q*_) to perform multi-class predictions with the weighted code prediction rule, y^
 MathType@MTEF@5@5@+=feaafiart1ev1aaatCvAUfKttLearuWrP9MDH5MBPbIqV92AaeXatLxBI9gBaebbnrfifHhDYfgasaacH8akY=wiFfYdH8Gipec8Eeeu0xXdbba9frFj0=OqFfea0dXdd9vqai=hGuQ8kuc9pgc9s8qqaq=dirpe0xb9q8qiLsFr0=vr0=vr0dc8meaabaqaciaacaGaaeqabaqabeGadaaakeaacuWG5bqEgaqcaaaa@2E37@ = arg max_*j*_(W * f→
 MathType@MTEF@5@5@+=feaafiart1ev1aaatCvAUfKttLearuWrP9MDH5MBPbIqV92AaeXatLxBI9gBaebbnrfifHhDYfgasaacH8akY=wiFfYdH8Gipec8Eeeu0xXdbba9frFj0=OqFfea0dXdd9vqai=hGuQ8kuc9pgc9s8qqaq=dirpe0xb9q8qiLsFr0=vr0=vr0dc8meaabaqaciaacaGaaeqabaqabeGadaaakeaacuWGMbGzgaWcaaaa@2E13@(*x*))·**C**_*j*_, where W * f→
 MathType@MTEF@5@5@+=feaafiart1ev1aaatCvAUfKttLearuWrP9MDH5MBPbIqV92AaeXatLxBI9gBaebbnrfifHhDYfgasaacH8akY=wiFfYdH8Gipec8Eeeu0xXdbba9frFj0=OqFfea0dXdd9vqai=hGuQ8kuc9pgc9s8qqaq=dirpe0xb9q8qiLsFr0=vr0=vr0dc8meaabaqaciaacaGaaeqabaqabeGadaaakeaacuWGMbGzgaWcaaaa@2E13@(*x*) denotes component-wise multiplication. We learn **W **by a cross-validation set-up on the training set, using a ranking perceptron. The full methodology consists of five steps: (1) split the training data into 10 cross-validation sets; (2) learn fold- and superfamily-level detectors from the partitioned training set – performing fold recognition and superfamily recognition on the held-out cross-validation sets, thereby generating training data for code weight learning; (3) use the ranking perceptron algorithm for learning the optimal weighting of classifiers in code space; (4) re-train superfamily and fold detectors on the full training set; and (5) test on the final untouched test set. Figure 2 shows a summary of the above steps, and Figure 3 shows the cross-validation scheme for learning **W**.

### Benchmark results

#### Remote homology detection performance

For the remote homology detection data sets, where the test set consists of held-out protein families that belong to superfamilies represented in the training data, we evaluate performance both for the superfamily-level and fold-level prediction tasks. Results for multi-class superfamily and fold prediction are provided in Tables 1 and 2, respectively. We compare our adaptive codes method to PSI-BLAST, a standard homology detection method based on sequence alignment, as well as simple one-vs-all, sigmoid fitting, using various choices of code vectors. In addition to reporting classification loss and balanced loss results, we give "top 5" classification and balanced loss performance, which evaluates whether the correct class was found in the top 5 class predictions. The motivation for top 5 loss results is that a structural biologist might be willing to investigate a small number of false positives if it was likely that the list also contained the true structural class. We also report results based on the false detection (FDR) rate for each of the methods. We report the percentage of true positives recovered when we set the threshold such that *FDR *= *FP*/(*FP *+ *TP*) is equal to 0.01 or 0.05, where *FP*, *TN* and *TP* are the number of false positives, true negatives and true positives, respectively. Because these are multi-class methods, the real-valued output for each possible output label *y *and each test example *x *is considered an individual prediction (which is either right or wrong) and a single threshold is chosen over all predictions. A similar procedure is conducted for PSI-BLAST. Here, the motivation is to compare how well the methods detect true remote homologs or recognize true fold members at a high confidence level.

For the superfamily prediction task, we find that the adaptive codes method significantly outperforms one-vs-all both in terms of classification and balanced error, even when superfamily-only codes are used, and performance improves as more elements are added to the codes. We also note that sigmoid fitting gives substantially worse performance than one-vs-all for this task. When compared to the widely-used PSI-BLAST method, even simple one-vs-all outperforms PSI-BLAST strongly in terms of classification error and slightly in terms of balanced error; adaptive codes outperforms PSI-BLAST very strongly by both measures and also when considering "top 5" prediction performance. In terms of the detection rate at low false discovery rates (FDR = 1% or 5%), all SVM-based methods significantly outperform PSI-BLAST, and in particular at FDR = 5%, there is a trend of greater detection rates as the code length increases. For the fold prediction task, we use a different set of codes, including code elements corresponding to protein fold detectors. We observe a similar trend, with adaptive codes again beating one-vs-all with respect to classification and balanced loss when fold-only codes are used and performance continuing to improve as the length of the codes increases. The best result for adaptive codes is significantly better than PSI-BLAST. Finally, sigmoid fitting slightly degrades performance as compared to one-vs-all. At low false positive rates, we again observe that adaptive codes has a greater detection rates as code elements are added, and results for the longest codes are significantly better than one-vs-all. However, for this problem, unlike the superfamily prediction task, PSI-BLAST has better detection performance at low false positive rates than the adaptive codes method, showing that PSI-BLAST is doing relatively well at high confidence predictions even though the overall error rates are worse.

Overall, we observe that when the individual code elements are helpful, as seems to be the case in remote homology detection, our adaptive codes method can successfully improve performance by adding elements without overfitting.

#### Fold recognition results

For the more difficult fold recognition task, where the data set consists of held-out superfamilies from protein folds represented in the training data, we expect that code elements from subclasses (i.e. superfamilies and families) will provide less information, since protein sequences from different superfamilies in principle have no detectable sequence similarity.

Results for the fold recognition problem are provided in Table 3. Note first that the errors for PSI-BLAST, even for the top 5 fold predictions, are very high, underscoring the difficulty of the problem. Sigmoid fitting appears to slightly help reduce one-vs-all error in this case, though balanced error is unaffected. We find that the adaptive codes method can again beat one-vs-all and strongly outperform PSI-BLAST in terms of prediction accuracy, but we see no trend of improvement as more code elements are added, with various length codes leading to similar error rates. At low false discovery rates, the adaptive codes method has much higher fold recognition rates than PSI-BLAST and also outperforms one-vs-all. Interestingly, here the best high confidence detection rate does occur for the longest codes, though the trend is unclear. We conclude that in this case, since the code elements corresponding to subclasses are not as helpful, the adaptive codes method cannot consistently leverage longer codes to achieve much higher accuracy. However, the weight learning approach does significantly outperform one-vs-all and greatly outperform PSI-BLAST by all evaluation measures.

### The SVM-Fold web server

**Figure 4:**
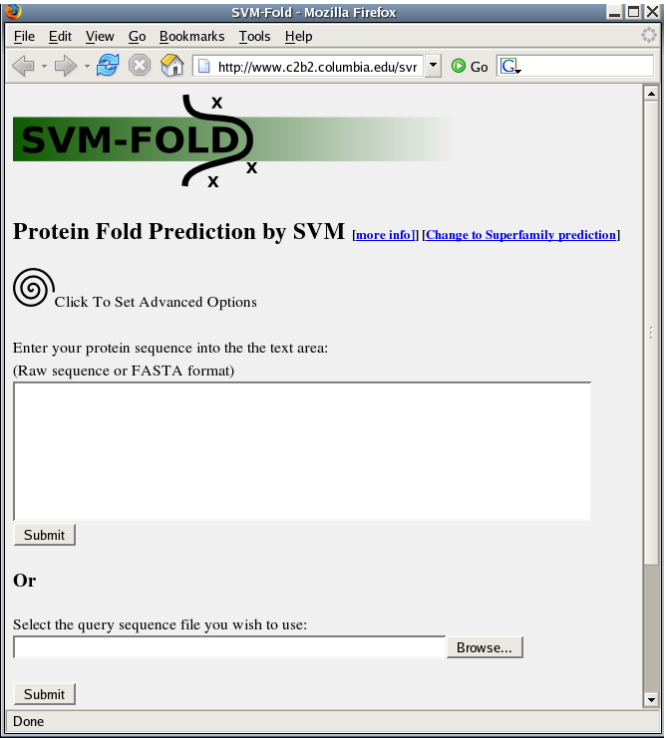
**SVM-Fold home page**. A screen capture of the SVM-Fold home page.

In this section, we describe some details of the web server that implements our system, which is available at SVM-Fold [15]. The server can perform both superfamily and fold detection but performs fold detection by default. One can switch between the two modes using a link on the front page. The coverage for each mode, that is, the set of folds or superfamilies that a given query sequence is ranked against, is given via a link named "coverage" on the front page. Users of the website can enter raw sequence data in FASTA format data directly into a form on the web-page, or select a local FASTA file to upload to the server (see Figure 4). Alternatively, the user can supply a PSI-BLAST profile file (output of blastpgp -Q) instead. In the advanced options on the main page one can select either zero-one or balanced loss optimized SVMs, or alternatively just use standard PSI-BLAST ranking.

**Figure 5:**
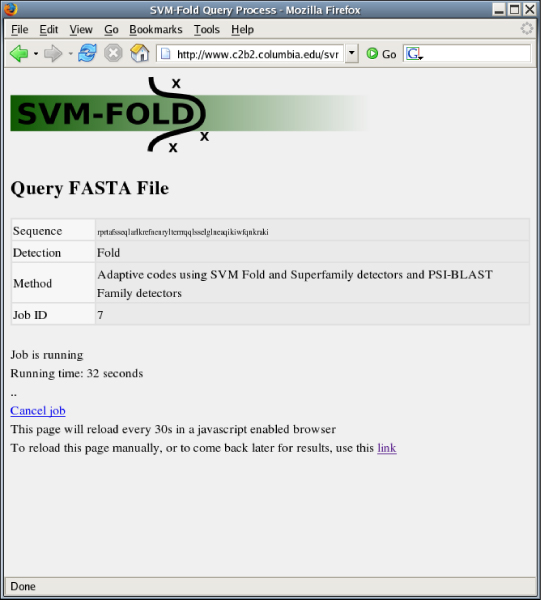
**SVM-Fold processing page**. A screen capture of the SVM-Fold processing page.

**Figure 6:**
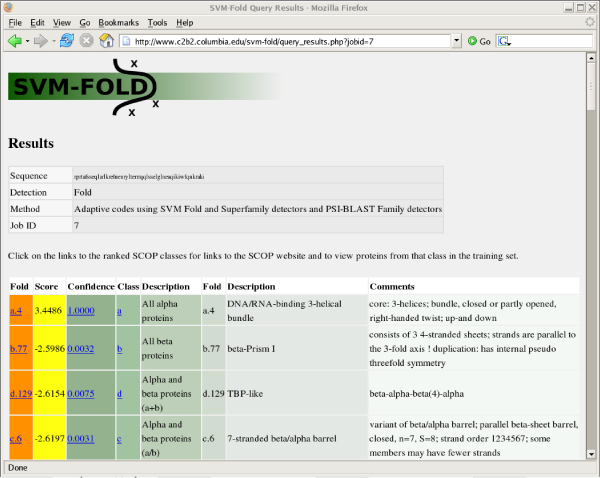
**SVM-Fold results page**. A screen capture of the SVM-Fold results page.

**Figure 7:**
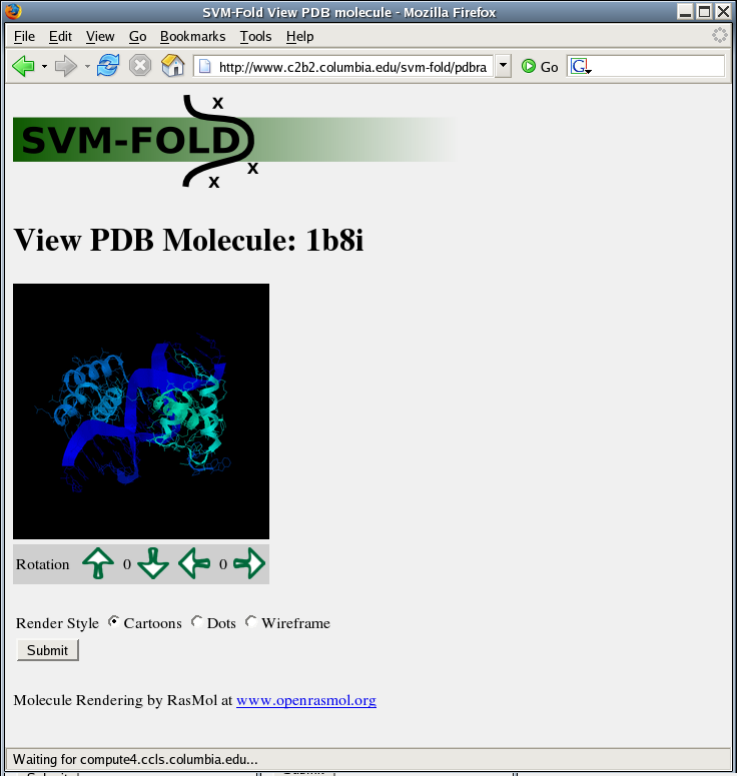
**SVM-Fold molecule view**. SVM-Fold provides links to views of representative 3D structures.

The server will then add the query to a queue and compute the results (See Figure 5). A query currently takes approximately 6 minutes from when it starts processing on a 2 Ghz AMD Opteron processor with 8 GB memory. Approximately 5 minutes of this computation is actually computing the profile using PSI-BLAST for use with the profile kernel. On completion, the user is presented with a table showing the resulting SCOP-Fold ranking of their sequence along with an empirically estimated confidence value (see Methods) and SCOP derived comments relating to the fold or superfamily (See Figure 6). The results table also contains links to pages detailing results for each target SCOP class. These pages link to the relevant pages on the SCOP website and show both PSI-BLAST E-values and profile kernel scores (see Methods) between the query protein and the set of proteins from the SCOP class in the training set. For each protein in these rankings, we can go to a full SCOP definition or to a molecule rendering of that protein. The molecule renderer uses OpenRasMol on the server-side to deliver small animated 3D renders, without the need for a browser plugin. There are controls on this page to rotate the molecule and to alter the render style (see Figure 7).

For fold detection, the website currently ranks a list of 65 SCOP folds, using detectors for 65 folds, 560 superfamilies and 1126 families. For superfamily detection, the website ranks a list of 174 SCOP superfamilies, using detectors for 174 superfamilies and 1036 families.

## Conclusion

In this article, we have described the fielded protein superfamily and fold recognition system, SVM-Fold. SVM-Fold uses the discriminative support vector machine algorithm with a state-of-the-art string kernel based on PSI-BLAST profiles to leverage unlabeled data. Binary one-vs-the-rest SVM classifiers that are trained to recognize individual structural classes yield prediction scores that are incomparable, so that standard "one-vs-all" classification performs suboptimally when the number of classes is very large, as in this case. To deal with this challenging problem, we have developed an adaptive multi-class codes algorithm that learns relative weights between one-vs-the-rest classifiers and, further, encodes information about the protein structural hierarchy for multi-class prediction. In large-scale benchmark results based on the SCOP database, our system significantly improves on the prediction accuary of both a baseline use of PSI-BLAST and the standard one-vs-all method on every structure classification problem we consider. The SVM-Fold web server now makes a state-of-the-art SVM-based protein classification system available to the bioinformatics community. In future work, we plan to increase the coverage of SCOP folds and superfamilies represented in our system and eventually extend our system to other structural taxonomies, such as CATH [26]. We will also develop and implement discriminative approaches to the problem of segmenting multi-domain protein sequences into domains by using SVM-based classifiers as domain recognizers.

## Methods

### Profile-based string kernel SVM

As a base for our multi-class protein classifier, we use profile-based string kernel SVMs [11] that are trained to perform binary classifications on the fold and superfamily levels of SCOP. The profile kernel is a function that measures the similarity of two protein sequence profiles based on their representation in a high-dimensional vector space indexed by all *k*-mers (*k*-length subsequences of amino acids). Specifically, for a sequence *x *and its sequence profile *P*(*x*) (e.g. PSI-BLAST profile), the *positional mutation *neighborhood is defined by the corresponding block of the profile *P*(*x*):

M(k,σ)(P(x[j+1:j+k]))={β=b1b2...bk:−∑i=1klog⁡pj+i(bi)<σ}.
 MathType@MTEF@5@5@+=feaafiart1ev1aaatCvAUfKttLearuWrP9MDH5MBPbIqV92AaeXatLxBI9gBaebbnrfifHhDYfgasaacH8akY=wiFfYdH8Gipec8Eeeu0xXdbba9frFj0=OqFfea0dXdd9vqai=hGuQ8kuc9pgc9s8qqaq=dirpe0xb9q8qiLsFr0=vr0=vr0dc8meaabaqaciaacaGaaeqabaqabeGadaaakeaacqWGnbqtdaWgaaWcbaGaeiikaGIaem4AaSMaeiilaWccciGae83WdmNaeiykaKcabeaakiabcIcaOiabdcfaqjabcIcaOiabdIha4jabcUfaBjabdQgaQjabgUcaRiabigdaXiabcQda6iabdQgaQjabgUcaRiabdUgaRjabc2faDjabcMcaPiabcMcaPiabg2da9iabcUha7jab=j7aIjabg2da9iabdkgaInaaBaaaleaacqaIXaqmaeqaaOGaemOyai2aaSbaaSqaaiabikdaYaqabaGccqGGUaGlcqGGUaGlcqGGUaGlcqWGIbGydaWgaaWcbaGaem4AaSgabeaakiabcQda6iabgkHiTmaaqahabaGagiiBaWMaei4Ba8Maei4zaCMaemiCaa3aaSbaaSqaaiabdQgaQjabgUcaRiabdMgaPbqabaGccqGGOaakcqWGIbGydaWgaaWcbaGaemyAaKgabeaakiabcMcaPaWcbaGaemyAaKMaeyypa0JaeGymaedabaGaem4AaSganiabggHiLdGccqGH8aapcqWFdpWCcqGG9bqFcqGGUaGlaaa@6F9F@

Note that the emission probabilities, *p*_*j*+*i*_(*b*), *i *= 1...*k*, come from the profile *P*(*x*) – for notational simplicity, we do not explicitly indicate the dependence on *x *. Typically, the profiles are estimated from close homologs found in a large sequence database; however, these estimates may be too restrictive for our purposes. Therefore, we smooth the estimates using background frequencies, *q*(*b*), *b *∈ Σ, of amino acids in the training data set via

p˜i(b)=pi(b)+tq(b)1+t,i=1...|x|,
 MathType@MTEF@5@5@+=feaafiart1ev1aaatCvAUfKttLearuWrP9MDH5MBPbIqV92AaeXatLxBI9gBaebbnrfifHhDYfgasaacH8akY=wiFfYdH8Gipec8Eeeu0xXdbba9frFj0=OqFfea0dXdd9vqai=hGuQ8kuc9pgc9s8qqaq=dirpe0xb9q8qiLsFr0=vr0=vr0dc8meaabaqaciaacaGaaeqabaqabeGadaaakeaacuWGWbaCgaacamaaBaaaleaacqWGPbqAaeqaaOGaeiikaGIaemOyaiMaeiykaKIaeyypa0ZaaSaaaeaacqWGWbaCdaWgaaWcbaGaemyAaKgabeaakiabcIcaOiabdkgaIjabcMcaPiabgUcaRiabdsha0jabdghaXjabcIcaOiabdkgaIjabcMcaPaqaaiabigdaXiabgUcaRiabdsha0baacqGGSaalcqWGPbqAcqGH9aqpcqaIXaqmcqGGUaGlcqGGUaGlcqGGUaGldaabdaqaaiabdIha4bGaay5bSlaawIa7aiabcYcaSaaa@501B@

where *t *is a smoothing parameter. We use the smoothed emission probabilities p˜
 MathType@MTEF@5@5@+=feaafiart1ev1aaatCvAUfKttLearuWrP9MDH5MBPbIqV92AaeXatLxBI9gBaebbnrfifHhDYfgasaacH8akY=wiFfYdH8Gipec8Eeeu0xXdbba9frFj0=OqFfea0dXdd9vqai=hGuQ8kuc9pgc9s8qqaq=dirpe0xb9q8qiLsFr0=vr0=vr0dc8meaabaqaciaacaGaaeqabaqabeGadaaakeaacuWGWbaCgaacaaaa@2E24@_*i*_(*b*) in place of *p*_*i*_(*b*) in defining the mutation neighborhoods.

We now define the profile feature mapping as

Φ(k,σ)Profile(P(x))=∑j=0...|x|−k(φβ(P(x[j+1:j+k])))β∈Σk
 MathType@MTEF@5@5@+=feaafiart1ev1aaatCvAUfKttLearuWrP9MDH5MBPbIqV92AaeXatLxBI9gBaebbnrfifHhDYfgasaacH8akY=wiFfYdH8Gipec8Eeeu0xXdbba9frFj0=OqFfea0dXdd9vqai=hGuQ8kuc9pgc9s8qqaq=dirpe0xb9q8qiLsFr0=vr0=vr0dc8meaabaqaciaacaGaaeqabaqabeGadaaakeaacqqHMoGrdaqhaaWcbaGaeiikaGIaem4AaSMaeiilaWccciGae83WdmNaeiykaKcabaacbaGae4huaaLae4NCaiNae43Ba8Mae4NzayMae4xAaKMae4hBaWMae4xzaugaaOGaeiikaGIaemiuaaLaeiikaGIaemiEaGNaeiykaKIaeiykaKIaeyypa0ZaaabuaeaacqGGOaakcqWFgpGzdaWgaaWcbaGae8NSdigabeaakiabcIcaOiabdcfaqjabcIcaOiabdIha4jabcUfaBjabdQgaQjabgUcaRiabigdaXiabcQda6iabdQgaQjabgUcaRiabdUgaRjabc2faDjabcMcaPiabcMcaPiabcMcaPmaaBaaaleaacqWFYoGycqGHiiIZcqqHJoWudaahaaadbeqaaiabdUgaRbaaaSqabaaabaGaemOAaOMaeyypa0JaeGimaaJaeiOla4IaeiOla4IaeiOla4YaaqWaaeaacqWG4baEaiaawEa7caGLiWoacqGHsislcqWGRbWAaeqaniabggHiLdaaaa@6F56@

where the coordinate *ϕ*_*β*_(*P*(*x*[*j *+ 1 : *j *+ *k*])) = 1 if *β *belongs to the mutation neighborhood *M*_(*k*,*σ*)_(*P*(*x*[*j *+ 1 : *j *+ *k*])), and otherwise the coordinate is 0. Note that the profile kernel between two protein sequences is simply defined by the inner product of feature vectors:

K(k,σ)Profile(P(x),P(y))=〈Φ(k,σ)Profile(P(x)),Φ(k,σ)Profile(P(y))〉.
 MathType@MTEF@5@5@+=feaafiart1ev1aaatCvAUfKttLearuWrP9MDH5MBPbIqV92AaeXatLxBI9gBaebbnrfifHhDYfgasaacH8akY=wiFfYdH8Gipec8Eeeu0xXdbba9frFj0=OqFfea0dXdd9vqai=hGuQ8kuc9pgc9s8qqaq=dirpe0xb9q8qiLsFr0=vr0=vr0dc8meaabaqaciaacaGaaeqabaqabeGadaaakeaacqWGlbWsdaqhaaWcbaGaeiikaGIaem4AaSMaeiilaWccciGae83WdmNaeiykaKcabaacbaGae4huaaLae4NCaiNae43Ba8Mae4NzayMae4xAaKMae4hBaWMae4xzaugaaOGaeiikaGIaemiuaaLaeiikaGIaemiEaGNaeiykaKIaeiilaWIaemiuaaLaeiikaGIaemyEaKNaeiykaKIaeiykaKIaeyypa0ZaaaWaaeaacqqHMoGrdaqhaaWcbaGaeiikaGIaem4AaSMaeiilaWIae83WdmNaeiykaKcabaGae4huaaLae4NCaiNae43Ba8Mae4NzayMae4xAaKMae4hBaWMae4xzaugaaOGaeiikaGIaemiuaaLaeiikaGIaemiEaGNaeiykaKIaeiykaKIaeiilaWIaeuOPdy0aa0baaSqaaiabcIcaOiabdUgaRjabcYcaSiab=n8aZjabcMcaPaqaaiab+bfaqjab+jhaYjab+9gaVjab+zgaMjab+LgaPjab+XgaSjab+vgaLbaakiabcIcaOiabdcfaqjabcIcaOiabdMha5jabcMcaPiabcMcaPaGaayzkJiaawQYiaiabc6caUaaa@7A24@

The use of profile-based string kernels is an example of *semi-supervised learning*, since unlabeled data in the form of a large sequence database is used in the discrimination problem. Moreover, profile kernel values can be efficiently computed in time that scales linearly with input sequence length. Equipped with such a kernel mapping, one can use SVMs to perform binary protein classification on the fold level and superfamily level. We call these trained SVMs *fold detectors *and *superfamily detectors*.

### Adaptive code-learning

Suppose that we have trained *q *fold detectors. Then for a protein sequence, *x*, we form a prediction discriminant vector, f→
 MathType@MTEF@5@5@+=feaafiart1ev1aaatCvAUfKttLearuWrP9MDH5MBPbIqV92AaeXatLxBI9gBaebbnrfifHhDYfgasaacH8akY=wiFfYdH8Gipec8Eeeu0xXdbba9frFj0=OqFfea0dXdd9vqai=hGuQ8kuc9pgc9s8qqaq=dirpe0xb9q8qiLsFr0=vr0=vr0dc8meaabaqaciaacaGaaeqabaqabeGadaaakeaacuWGMbGzgaWcaaaa@2E13@(*x*) = (*f*_1_(*x*),...,*f*_*q*_(*x*)). The simple one-versus all prediction rule for multi-class fold recognition is y^
 MathType@MTEF@5@5@+=feaafiart1ev1aaatCvAUfKttLearuWrP9MDH5MBPbIqV92AaeXatLxBI9gBaebbnrfifHhDYfgasaacH8akY=wiFfYdH8Gipec8Eeeu0xXdbba9frFj0=OqFfea0dXdd9vqai=hGuQ8kuc9pgc9s8qqaq=dirpe0xb9q8qiLsFr0=vr0=vr0dc8meaabaqaciaacaGaaeqabaqabeGadaaakeaacuWG5bqEgaqcaaaa@2E37@ = arg max_*j *_*f*_*j*_(*x*). The primary problem with this prediction rule is that the discriminant values produced by the different SVM classifiers are not necessarily comparable. While methods have been proposed to convert SVM discriminant scores into probabilistic outputs, for example using sigmoid fitting [22], in practice there may be insufficient data to estimate the sigmoid, or the fit may be poor. Our approach, in contrast, is to learn the optimal weighting for a set of classifiers, thereby scaling their discriminant values and making them more readily comparable. We also incorporate information available from the superfamily detectors for doing multi-class superfamily and fold recognition by designing output codes.

We construct our codes to incorporate knowledge about the known structural hierarchy provided by SCOP. Define for superfamily classes *j *∈ {1,...,*k*}, code vectors **C**_*j *_= (superfam_*j*_, fold_*j*_), where superfam_*j *_and fold_*j *_are vectors with length equal to the number of known superfamilies (*k*) and folds (*q*), and each of these two vectors has exactly one non-zero component corresponding to structural class identity. Each component in **C**_*j *_is known as a *code element *and represents the discriminant value given by the corresponding classifier.

We adapt the coding system to fit the training data by learning a weighting of the code elements (or classifiers). The final multi-class prediction rule is y^
 MathType@MTEF@5@5@+=feaafiart1ev1aaatCvAUfKttLearuWrP9MDH5MBPbIqV92AaeXatLxBI9gBaebbnrfifHhDYfgasaacH8akY=wiFfYdH8Gipec8Eeeu0xXdbba9frFj0=OqFfea0dXdd9vqai=hGuQ8kuc9pgc9s8qqaq=dirpe0xb9q8qiLsFr0=vr0=vr0dc8meaabaqaciaacaGaaeqabaqabeGadaaakeaacuWG5bqEgaqcaaaa@2E37@ = arg max_*j*_(W * f→
 MathType@MTEF@5@5@+=feaafiart1ev1aaatCvAUfKttLearuWrP9MDH5MBPbIqV92AaeXatLxBI9gBaebbnrfifHhDYfgasaacH8akY=wiFfYdH8Gipec8Eeeu0xXdbba9frFj0=OqFfea0dXdd9vqai=hGuQ8kuc9pgc9s8qqaq=dirpe0xb9q8qiLsFr0=vr0=vr0dc8meaabaqaciaacaGaaeqabaqabeGadaaakeaacuWGMbGzgaWcaaaa@2E13@(*x*))·**C**_*j*_, where * denotes the component-wise multiplication between vectors. To learn the weight vector **W**, we formulate a hard margin optimization problem as min⁡W‖W‖22
 MathType@MTEF@5@5@+=feaafiart1ev1aaatCvAUfKttLearuWrP9MDH5MBPbIqV92AaeXatLxBI9gBaebbnrfifHhDYfgasaacH8akY=wiFfYdH8Gipec8Eeeu0xXdbba9frFj0=OqFfea0dXdd9vqai=hGuQ8kuc9pgc9s8qqaq=dirpe0xb9q8qiLsFr0=vr0=vr0dc8meaabaqaciaacaGaaeqabaqabeGadaaakeaacyGGTbqBcqGGPbqAcqGGUbGBdaWgaaWcbaacbeGae83vaCfabeaakmaafmaabaGae83vaCfacaGLjWUaayPcSdWaa0baaSqaaiabikdaYaqaaiabikdaYaaaaaa@38AC@, subject to (W * f→
 MathType@MTEF@5@5@+=feaafiart1ev1aaatCvAUfKttLearuWrP9MDH5MBPbIqV92AaeXatLxBI9gBaebbnrfifHhDYfgasaacH8akY=wiFfYdH8Gipec8Eeeu0xXdbba9frFj0=OqFfea0dXdd9vqai=hGuQ8kuc9pgc9s8qqaq=dirpe0xb9q8qiLsFr0=vr0=vr0dc8meaabaqaciaacaGaaeqabaqabeGadaaakeaacuWGMbGzgaWcaaaa@2E13@ (*x*_*i*_))·(**C**_*y*_*i*__ - **C**_*j*_) ≥ 1, ∀*j *≠ *y*_*i*_.

Intuitively, our problem is to find an optimal re-weighting of the discriminant vector elements such that a weighted embedding of the discriminant vector in code space *R *^*k*+*q *^will exhibit large margins to discriminate between correct and incorrect codes (i.e., class identity).

**Figure 8:**
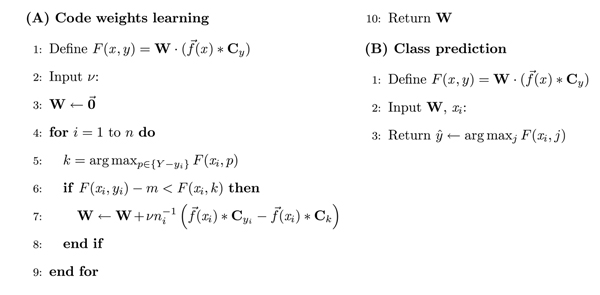
**Pseudocode for the ranking perceptron algorithm used to learn code weighting**. In the pseudocode, *ν *is the learning rate; *n*_*i *_= |{*y*_*j *_: *y*_*j *_= *y*_*i*_}| for balanced-loss, and *n*_*i *_= 1 for zero-one loss.

The ranking perceptron algorithm [27] is a variant of the well-known perceptron linear classifier [28]. In our experiments, the ranking perceptron receives as input the discriminant vectors for training sequences and produces as output a weight vector **W **which is a linear combination of the input vectors projected onto code space. We modify the ranking perceptron algorithm such that it will learn our weight vector **W **by satisfying *n *constraints:

**W**·(f→
 MathType@MTEF@5@5@+=feaafiart1ev1aaatCvAUfKttLearuWrP9MDH5MBPbIqV92AaeXatLxBI9gBaebbnrfifHhDYfgasaacH8akY=wiFfYdH8Gipec8Eeeu0xXdbba9frFj0=OqFfea0dXdd9vqai=hGuQ8kuc9pgc9s8qqaq=dirpe0xb9q8qiLsFr0=vr0=vr0dc8meaabaqaciaacaGaaeqabaqabeGadaaakeaacuWGMbGzgaWcaaaa@2E13@(*x*_*i*_) * Cyi
 MathType@MTEF@5@5@+=feaafiart1ev1aaatCvAUfKttLearuWrP9MDH5MBPbIqV92AaeXatLxBI9gBaebbnrfifHhDYfgasaacH8akY=wiFfYdH8Gipec8Eeeu0xXdbba9frFj0=OqFfea0dXdd9vqai=hGuQ8kuc9pgc9s8qqaq=dirpe0xb9q8qiLsFr0=vr0=vr0dc8meaabaqaciaacaGaaeqabaqabeGadaaakeaaieqacqWFdbWqdaWgaaWcbaGaemyEaK3aaSbaaWqaaiabdMgaPbqabaaaleqaaaaa@30FB@ - f→
 MathType@MTEF@5@5@+=feaafiart1ev1aaatCvAUfKttLearuWrP9MDH5MBPbIqV92AaeXatLxBI9gBaebbnrfifHhDYfgasaacH8akY=wiFfYdH8Gipec8Eeeu0xXdbba9frFj0=OqFfea0dXdd9vqai=hGuQ8kuc9pgc9s8qqaq=dirpe0xb9q8qiLsFr0=vr0=vr0dc8meaabaqaciaacaGaaeqabaqabeGadaaakeaacuWGMbGzgaWcaaaa@2E13@(*x*_*i*_) * **C**_*j*_) ≥ *m*, ∀*j *≠ *y*_*i *_    (1)

where *m *is the size of the margin we enforce (Figure 8).

The update rule of the ranking perceptron algorithm can be different depending on what kind of loss function one is aiming to optimize. In standard *zero-one loss *(or classification loss), one counts all prediction mistakes equally,

lz(y,y^)={1ify^≠y;0otherwise.
 MathType@MTEF@5@5@+=feaafiart1ev1aaatCvAUfKttLearuWrP9MDH5MBPbIqV92AaeXatLxBI9gBaebbnrfifHhDYfgasaacH8akY=wiFfYdH8Gipec8Eeeu0xXdbba9frFj0=OqFfea0dXdd9vqai=hGuQ8kuc9pgc9s8qqaq=dirpe0xb9q8qiLsFr0=vr0=vr0dc8meaabaqaciaacaGaaeqabaqabeGadaaakeaacqWGSbaBdaWgaaWcbaGaemOEaOhabeaakiabcIcaOiabdMha5jabcYcaSiqbdMha5zaajaGaeiykaKIaeyypa0ZaaiqaaeaafaqaaaGacaaabaGaeGymaedabaacbaGae8xAaKMae8NzayMae8hiaaIafmyEaKNbaKaacqGHGjsUcqWG5bqEcqGG7aWoaeaacqaIWaamaeaacqWFVbWBcqWF0baDcqWFObaAcqWFLbqzcqWFYbGCcqWF3bWDcqWFPbqAcqWFZbWCcqWFLbqzcqGGUaGlaaaacaGL7baaaaa@4FEA@

The final zero-one empirical loss is 1n∑ilz(yi,y^i)
 MathType@MTEF@5@5@+=feaafiart1ev1aaatCvAUfKttLearuWrP9MDH5MBPbIqV92AaeXatLxBI9gBaebbnrfifHhDYfgasaacH8akY=wiFfYdH8Gipec8Eeeu0xXdbba9frFj0=OqFfea0dXdd9vqai=hGuQ8kuc9pgc9s8qqaq=dirpe0xb9q8qiLsFr0=vr0=vr0dc8meaabaqaciaacaGaaeqabaqabeGadaaakeaadaWcaaqaaiabigdaXaqaaiabd6gaUbaadaaeqaqaaiabdYgaSnaaBaaaleaacqWG6bGEaeqaaOGaeiikaGIaemyEaK3aaSbaaSqaaiabdMgaPbqabaGccqGGSaalcuWG5bqEgaqcamaaBaaaleaacqWGPbqAaeqaaOGaeiykaKcaleaacqWGPbqAaeqaniabggHiLdaaaa@3E1D@. In *balanced loss*, the cost of each mistake is inversely proportional to the true class size,

lb(y,y^)={1|yi:yi=y|ify^≠y;0otherwise.
 MathType@MTEF@5@5@+=feaafiart1ev1aaatCvAUfKttLearuWrP9MDH5MBPbIqV92AaeXatLxBI9gBaebbnrfifHhDYfgasaacH8akY=wiFfYdH8Gipec8Eeeu0xXdbba9frFj0=OqFfea0dXdd9vqai=hGuQ8kuc9pgc9s8qqaq=dirpe0xb9q8qiLsFr0=vr0=vr0dc8meaabaqaciaacaGaaeqabaqabeGadaaakeaacqWGSbaBdaWgaaWcbaGaemOyaigabeaakiabcIcaOiabdMha5jabcYcaSiqbdMha5zaajaGaeiykaKIaeyypa0ZaaiqaaeaafaqaaaGacaaabaWaaSaaaeaacqaIXaqmaeaadaabdaqaaiabdMha5naaBaaaleaacqWGPbqAaeqaaOGaeiOoaOJaemyEaK3aaSbaaSqaaiabdMgaPbqabaGccqGH9aqpcqWG5bqEaiaawEa7caGLiWoaaaaabaacbaGae8xAaKMae8NzayMae8hiaaIafmyEaKNbaKaacqGHGjsUcqWG5bqEcqGG7aWoaeaacqaIWaamaeaacqWFVbWBcqWF0baDcqWFObaAcqWFLbqzcqWFYbGCcqWF3bWDcqWFPbqAcqWFZbWCcqWFLbqzcqGGUaGlaaaacaGL7baaaaa@5C81@

The final balanced empirical loss is 1|Y|∑ilb(yi,y^i)
 MathType@MTEF@5@5@+=feaafiart1ev1aaatCvAUfKttLearuWrP9MDH5MBPbIqV92AaeXatLxBI9gBaebbnrfifHhDYfgasaacH8akY=wiFfYdH8Gipec8Eeeu0xXdbba9frFj0=OqFfea0dXdd9vqai=hGuQ8kuc9pgc9s8qqaq=dirpe0xb9q8qiLsFr0=vr0=vr0dc8meaabaqaciaacaGaaeqabaqabeGadaaakeaadaWcaaqaaiabigdaXaqaamaaemaabaGaemywaKfacaGLhWUaayjcSdaaamaaqababaGaemiBaW2aaSbaaSqaaiabdkgaIbqabaGccqGGOaakcqWG5bqEdaWgaaWcbaGaemyAaKgabeaakiabcYcaSiqbdMha5zaajaWaaSbaaSqaaiabdMgaPbqabaGccqGGPaqkaSqaaiabdMgaPbqab0GaeyyeIuoaaaa@40E5@, where *Y *denotes the set of output labels.

Balanced loss is relevant to protein structure prediction because class sizes are unbalanced, and we do not want to perform well only on the largest classes. The particular ranking perceptron training and prediction algorithms that we use are summarized in the pseudocode in Figure 8, including update rules for both zero-one and balanced loss.

#### Relation to other approaches

We note that Rätsch et al. [29] considered a more general and difficult problem of adapting codes and embeddings, that is, learning both the code vectors and the embedding of the vector of prediction scores in output space via a non-convex optimization problem. In addition, Crammer and Singer [20] formulated another more general problem of learning a mapping of all inputs to all outpus. By restricting ourselves to the simpler problem of reweighting the output space so that our fixed codes perform well, we are able to define a convex large-margin optimization problem that can be efficiently solved with recent methods and is tractable in very large scale settings. Unlike the other two approaches, we can also choose which loss function we wish to optimize – for example, in protein classification, we can use the balanced loss, so that performance on the large classes does not dominate the results – and we can make use of hierarchical labels.

### Confidence scores

**Figure 9:**
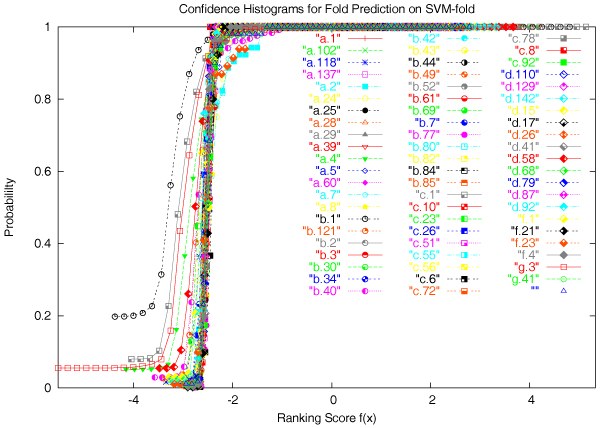
**SVM-Fold fold detection confidence**. Confidence vs. ranking score for fold detection on the SVM-Fold server.

In the results table for the webserver, we also provide a confidence score for each target class, which is the empirical probability that the ranking score is correct. This is achieved by performing a cross-validation on the training set and and creating a histogram of probabilities for each class given the ranking scores and their correct labels. These are shown in Figure 9.

### Optimizations

The profile kernel for the entire training set can be efficiently computed using a trie data structure [11]. To do this, one performs a lexical traversal of all instances of *k*-mers that appear in the dataset. Hence, the tree is a rooted tree of depth *k *where each internal node has 20 branches, each labeled with an amino acid. A leaf node represents a fixed *k*-mer in our feature space, obtained by concatenating branch symbols along the path from root to leaf. We perform a depth-first traversal of the data structure, and at each step we maintain the list of *k*-mers, and the proteins that they are derived from, that appear in the training set which are in the positional mutation neighborhood of the current node in the tree. Each passage from parent node to child node reduces the size of this list; as soon as this list is empty, further branches from this node are not explored. If a leaf node is reached we can update the kernel matrix using the remaining *k*-mers in the list.

The profile kernel method employed by the webserver is optimized to produce one row of the kernel matrix. This is implemented as above except one only needs to traverse nodes that contain *k*-mers from the query sequence. A further refinement is that the webserver caches the tree of *k*-mers found in the training dataset to the first three levels of the tree, taking approximately 8 GB of memory. On receving a query, the server process forks and completes the tree with respect to the query. Caching the tree reduced the profile kernel portion of the query processing time in a typical example from 3 min 43 s to 1 min 18 s.

## Authors' contributions

IM implemented the webserver, SVM-Fold, conducted the computer experiments described here, and helped draft the manuscript. EI conducted the earlier research and experiments described in the conference version of this work. RK developed the profile kernel SVM which we use for SVM-Fold. JW helped develop the adaptive code technique. WSN and CL coordinated the research, helped design the experiments and draft the manuscript.

### Competing interests

The authors declare that they have no competing interests.

## Figures and Tables

**Table 1:** Results for multi-class superfamily prediction in the remote homology detection set-up. Results for the adaptive codes method are reported for a SCOP benchmark data set (67 folds, 74 superfamilies, 544 families, with 802 test sequences).

Method (and optimization target)	Error	Balanced Error	Top 5 Error	Balanced Top 5 Error	Detection Rate at fdr = 1%	Detection Rate at fdr = 5%
PSI-BLAST	0.3990	0.4571	0.2731	0.3654	0.3229	0.4214
one-vs-all: Sfams	0.2706	0.4454	0.1047	0.1973	0.4239	0.5549
one-vs-all: Sfams, Fams	0.2706	0.4454	0.1097	0.2070	0.4239	0.5549
Sigmoid Fitting: Sfams	0.3678	0.5561	0.2020	0.3724	0.3641	0.4726
Adaptive Codes: Sfams (zero-one)	0.2444	0.3805	0.0960	0.1591	0.4264	0.5711
Adaptive Codes: Sfams (balanced)	0.2481	0.3723	0.1110	0.1634	0.4289	0.5673
Adaptive Codes: Sfams, Fams (zero-one)	0.2369	0.3739	0.0948	0.1561	0.4352	0.5698
Adaptive Codes: Sfams, Fams (balanced)	0.2394	0.3632	0.1047	0.1558	0.4302	0.5698
Adaptive Codes: Sfams, Fams, Fams (zero-one)	0.2219	0.3401	0.0910	0.1359	0.4277	0.5723
Adaptive Codes: Sfams, Fams, Fams (balanced)	0.2195	0.3273	0.1047	0.1516	0.4327	0.5910

**Table 2:** Results for multi-class fold prediction in the remote homology detection set-up. Results for the adaptive codes method are reported for a SCOP benchmark data set (44 folds, 424 superfamilies, 809 families, with 381 test sequences).

Method (and optimization target)	Error	Balanced Error	Top 5 Error	Balanced Top 5 Error	Detection Rate at fdr = 1%	Detection Rate at fdr = 5%
PSI-BLAST	0.4094	0.4428	0.2966	0.3666	0.3123	0.3412
one-vs-all: Folds	0.3307	0.4565	0.1260	0.1954	0.2073	0.2493
one-vs-all: Folds, Sfams	0.3307	0.4565	0.1260	0.1954	0.2073	0.2336
Sigmoid Fitting: Folds	0.3465	0.4973	0.1706	0.3407	0.2178	0.2283
Adaptive Codes: Folds (zero-one)	0.3018	0.3769	0.1286	0.1862	0.2073	0.2362
Adaptive Codes: Folds (balanced)	0.3281	0.3766	0.1680	0.1702	0.2073	0.2362
Adaptive Codes: Folds, Sfams (zero-one)	0.2808	0.3749	0.1155	0.1770	0.2283	0.2493
Adaptive Codes: Folds, Sfams (balanced)	0.2887	0.3659	0.1260	0.1427	0.2231	0.2388
Adaptive Codes: Folds, Sfams, Fams (zero-one)	0.2493	0.3474	0.1024	0.1726	0.2231	0.2677
Adaptive Codes: Folds, Sfams, Fams (balanced)	0.2703	0.3445	0.1155	0.1418	0.2283	0.2703

**Table 3:** Results for multi-class fold prediction in the fold recognition set-up. Results for the adaptive codes method are reported on a SCOP benchmark data set (26 folds, 303 superfamilies, 614 test sequences).

Method (and optimization target)	Error	Balanced Error	Top 5 Error	Balanced Top 5 Error	Recognition Rate at fdr = 1%	Recognition Rate at fdr = 5%
PSI-BLAST	0.6482	0.7029	0.5179	0.5431	0.0814	0.0961
one-vs-all: Folds	0.4625	0.6282	0.1450	0.2345	0.1368	0.2704
one-vs-all: Folds, Sfams	0.4625	0.6282	0.1450	0.2345	0.1368	0.2704
Sigmoid Fitting: Folds	0.4446	0.6103	0.1547	0.2960	0.1336	0.2166
Adaptive Codes: Folds (zero-one)	0.4023	0.5556	0.1059	0.1543	0.1906	0.2655
Adaptive Codes: Folds (balanced)	0.3664	0.5158	0.1075	0.1387	0.1612	0.2785
Adaptive Codes: Folds, Sfams (zero-one)	0.4104	0.5525	0.1107	0.1719	0.2003	0.2329
Adaptive Codes: Folds, Sfams (balanced)	0.3616	0.5153	0.1010	0.1263	0.2068	0.2508
Adaptive Codes: Folds, Sfams, Fams (zero-one)	0.4007	0.5427	0.1075	0.1788	0.2068	0.2557
Adaptive Codes: Folds, Sfams, Fams (balanced)	0.3648	0.5000	0.1091	0.1453	0.2134	0.3013
